# Mycotoxin-Induced Oxidative Stress and Its Impact on Human Folliculogenesis: Examining the Link to Reproductive Health

**DOI:** 10.3390/toxins17120574

**Published:** 2025-11-28

**Authors:** Zsuzsanna Szőke, Eszter Ruff, Patrik Plank, Zsófia Molnár, Lili Hruby, Apolka Szentirmay, Márkó Unicsovics, Bernadett Csókay, Katalin Varga, Tímea Buzder, Miklós Sipos, Katalin Sára-Popovics, Dóra Holéci, Katalin Posta, Levente Sára

**Affiliations:** 1 Agribiotechnology and Precision Breeding for Food Security National Laboratory, Department of Animal Biotechnology, Institute of Genetics and Biotechnology, Hungarian University of Agriculture and Life Sciences, 2100 Gödöllő, Hungary; plankpatrik94@gmail.com (P.P.); molnar.zsofia@uni-mate.hu (Z.M.); katalinsarapopovics@gmail.com (K.S.-P.); holeci.dora@uni-mate.hu (D.H.); 2 Department of Obstetrics and Gynecology, Semmelweis University, 1088 Budapest, Hungary; ruff.eszter@semmelweis.hu (E.R.); szentirmay.apolka@gmail.com (A.S.); u.marko92@gmail.com (M.U.); cspupi@gmail.com (B.C.); sara.levente@semmelweis.hu (L.S.); 3 Faculty of Medicine, University of Freiburg, 79110 Freiburg, Germany; lili.hruby@uniklinik-freiburg.de; 4 Central of Assisted Reproduction, Semmelweis University, 1097 Budapest, Hungary; vargakata2001@gmail.com (K.V.); buzder.timea@semmelweis.hu (T.B.); sipos.miklos@semmelweis.hu (M.S.); 5 Department of Chemistry, Institute of Mathematics and Basic Science, 2100 Gödöllő, Hungary; 6 Department of Microbiology and Applied Biotechnology, Institute of Genetics and Biotechnology, Hungarian University of Agriculture and Life Sciences, 2100 Gödöllő, Hungary

**Keywords:** mycotoxins, follicular fluid, oxidative stress, infertility, deoxynivalenol (DON), zearalenone (ZEN), anti-Müllerian hormone (AMH), estradiol, endocrine disruption, oocyte quality

## Abstract

Climate change has contributed to increased mycotoxin contamination in food systems, posing a growing threat to human health, including reproductive health. Our study aimed to investigate how mycotoxins entering the follicular fluid affect oxidative stress processes. We analyzed 88 follicular fluid samples from infertile patients for common mycotoxins, including deoxynivalenol (DON), zearalenone (ZEN), its main metabolite alpha-zearalenol (aZOL), and aflatoxin M1 (AfM1), and examined their relationship with oxidative stress markers (MDA, SOD, GPx, CAT, and TAOC) and hormones (cortisol, estradiol, and anti-Müllerian hormone). Higher mycotoxin levels were associated with increased oxidative stress, particularly elevated MDA levels, and disrupted antioxidant enzyme activity. Notably, DON showed a positive correlation with SOD and estradiol levels, indicating a compensatory antioxidant response, while AfM1 served as a negative predictor. The metabolite aZOL was strongly linked to cortisol, with effects influenced by estradiol levels, implying endocrine-disrupting activity. Importantly, the interaction between DON and AMH appeared to impact dominant follicle development, suggesting a potential mechanism by which environmental toxins impair fertility without directly reducing oocyte or embryo counts. These results highlight the complex, dose-dependent effects of mycotoxins on oxidative and hormonal balances within the follicular environment, with implications for oocyte quality and reproductive success. Better understanding these mechanisms could help develop early diagnostic markers and targeted interventions to improve fertility outcomes in women exposed to changing environmental conditions.

## 1. Introduction

The growing impact of climate change, characterized by rising temperatures and extreme weather events, is increasingly recognized as a significant factor in altering fungal behavior and distribution, thereby escalating the risk of human and animal exposure to mycotoxins [1]. These harmful compounds, produced by fungi, pose a significant threat to human and animal health by contaminating food and feed [2,3].

According to the latest WHO study [4], we are faced with a problem affecting one in six couples. However, the theory that infertility is a burden inextricably linked to urbanization, civilizational changes, and the increase in living standards appears to be falling apart, as there is no significant difference in rates between developed countries and the developing world (WHO). In addition to the well-known causes of infertility, in nearly 15–30% of cases, the underlying causes have not been clarified even today [5]. Mycotoxins are particularly concerning due to their impact on folliculogenesis, the process of ovarian follicle maturation that is crucial for oocyte development and ovulation [6].

Mycotoxins, including aflatoxins (AFs), zearalenone (ZEN) and its metabolites, deoxynivalenol (DON), fumonisins, and ochratoxin A (OTA), induce oxidative stress, which is an imbalance between reactive oxygen species (ROS) production and antioxidant defenses [7,8,9,10,11]. This imbalance can damage cellular components and disrupt critical biological processes [12]. Oxidative stress results in DNA damage, lipid peroxidation, and protein damage, which may ultimately lead to cell death. Mycotoxins intensify this stress, and this mechanism is a critical factor in their toxic effects [13].

Mycotoxins often trigger oxidative stress by disrupting mitochondrial function and altering the cellular redox status [14]. This oxidative imbalance is particularly detrimental to folliculogenesis, a process that is highly dependent on the meticulously balanced follicular fluid that provides a crucial microenvironment for oocyte development [15]. Mycotoxins can penetrate follicular fluid even at low serum levels, potentially causing localized disruption of follicular maturation processes. For instance, ZEN and its metabolites are known endocrine disruptors that interfere with hormonal signaling pathways essential for proper follicular development, thereby impacting estradiol and progesterone levels [6,8]. In reproductive cells, ZEN disrupts the development of follicles. Low concentrations stimulate the proliferation of equine granulosa cells, potentially leading to follicular atresia [16]. ZEN also negatively impacts the proliferation and genomic stability of porcine granulosa cells, contributing to reproductive issues [17]. ZEN, with its estrogen-like effects, can impair ovarian function and uterine health, thereby affecting follicular development and female fertility [16,17,18].

The combined effects of mycotoxins, oxidative stress, and hormonal disruption create a cascade of adverse events within the follicles. Mycotoxins, such as T-2 toxin, can reduce FSH-stimulated estradiol production and alter progesterone production in granulosa cells, extensively impacting hormone regulation during folliculogenesis [19]. Oxidative stress induced by mycotoxins exacerbates hormonal imbalances, compromising oocyte quality and potentially leading to infertility or pregnancy complications. Elevated ROS levels can damage follicular cellular components, alter the composition of follicular fluid, and negatively affect oocyte health.

Malondialdehyde (MDA), a biomarker of oxidative stress derived from lipid peroxidation, is elevated in response to mycotoxin exposure, indicating oxidative damage [10,20]. Superoxide dismutases (SODs) are crucial antioxidant enzymes that protect cells from oxidative stress by converting superoxide anions into less reactive substances [21]. SOD is present in human follicular fluid, with higher activity than in serum, highlighting its importance in the ovarian microenvironment [22,23]. Its distribution and activity vary with follicular development, with theca interna cells showing intensive SOD staining, suggesting a role in protecting oocytes from oxygen radicals during maturation [23]. Cortisol, a stress response glucocorticoid hormone, regulates metabolic processes. Elevated cortisol levels can impact oxidative stress pathways, potentially increasing MDA levels and modifying SOD activity. FF Cortisol also positively influences oocyte maturation, with higher follicular levels seen in mature oocytes [24]. The total antioxidant capacity (TAOC) of follicular fluid plays a crucial role in folliculogenesis and oocyte development, with higher levels correlating with improved pregnancy rates during in vitro fertilization (IVF) treatment [25]. TAOC protects oocytes from oxidative stress, potentially improving oocyte quality and reproductive potential [26,27]. A study in rats showed that exposure to a combination of mycotoxins—specifically fumonisin B1 (FB1), deoxynivalenol (DON), and zearalenone (ZEN)—increased both total glutathione and the activity of the enzyme glutathione peroxidase (GPx) in the liver. This effect was found to be synergistic, meaning the combined toxins enhanced GPx activity more than each toxin did individually [28]. In humans, GPx activity is known to increase in response to oxidative stress, as seen in non-insulin-dependent diabetic women [29], Alternariol (AOH) mycotoxin exposure led to a 30% reduction in glutathione peroxidase (GPx) activity in human colon cells [30]. This suggests that GPx plays an adaptive role in the body’s defense system, which would likely be triggered by toxic exposures, such as from mycotoxins. Catalase is a crucial enzyme that serves as a cellular defense mechanism against oxidative stress, which is a common consequence of toxic exposure, including that from mycotoxins. Catalase helps decompose hydrogen peroxide into water and oxygen, thereby preventing cellular damage from oxidative stress [31]. Mycotoxins such as aflatoxins, ochratoxins, and deoxynivalenol can lead to varied toxic effects, including oxidative stress, which can subsequently affect catalase activity in exposed tissues [32,33].

This study examines the complex relationship between mycotoxins, oxidative stress, and human folliculogenesis, emphasizing their potential impact on fertility. The presence and levels of specific mycotoxins in human follicular fluid, as well as oxidative stress biomarkers, such as MDA, SOD, TAOC, CAT, and GPx activity, were analyzed. It also investigates the possible connection between climate-driven mycotoxin-induced oxidative stress in follicular fluid and outcomes of human folliculogenesis, including oocyte maturation and overall reproductive health.

## 2. Results

### 2.1. Biophysical Parameters and Mycotoxin Levels in the Context of Follicle Number

**Figure 1:**
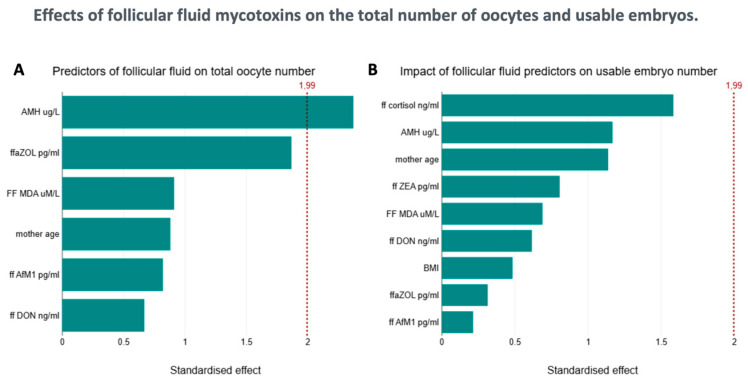
**Effects of follicular fluid mycotoxins on the total number of oocytes and usable embryos.** The Pareto diagrams display the relative effect and its significance (red line) on folliculogenesis. The AMH level is a significant positive predictor of the total number of oocytes obtained after stimulation and retrieval (**A**) ffaZOL concentration also has a positive effect on oocyte number, but it did not reach the level of significance. None of the factors examined proved to be significant regarding the number of usable embryos (**B**).

Eighty-eight follicular fluid samples were analyzed. The mean age of the patients was 36.44 (±0.42 SEM) years, and their body mass index (BMI) was 24.6 (±0.44 SEM). The mean AMH level was 2.092 ± 0.2 μg/L (Table 1). As we previously reported, all examined mycotoxins were detected in the follicular fluid [6]. Table 2 shows the mycotoxin levels measured in serum and follicular fluid and standardized to protein levels. Age, BMI, and AMH did not correlate with standardized levels of MDA, SOD, or cortisol concentrations in the follicular fluid. No correlation was found between the number of follicles or dominant follicles and the concentrations of almost all tested mycotoxins. However, we observed a positive trend between total and dominant follicle numbers and ffaZOL (LR: 5.8, *p* < 0.05, and LR: 7.54, *p* < 0.01), as well as between ffaZOL and the number of metaphase II oocytes (LR: 4.33, *p* < 0.05), after applying a Quasi-Poisson correction and a negative binomial transformation—though the results did not reach statistical significance (Figure 1).

Furthermore, no correlation was observed between the number of usable embryos, their ratio to total follicles, and mycotoxin levels. Our results identified age (F (1, 76) = 1.63, *p* = 0.206) as a non-significant negative factor, and AMH level (F (1, 76) = 33.3, *p* < 0.0001) a significant, strong positive factor influencing follicle count. We noted a significant interaction between DON and AMH (borderline in Quasi-Poisson correction); as the DON level decreases, the positive effect of AMH on the number of dominant follicles becomes more evident (LR (71): 14.73, *p*_LR: 1.24 × 10^−4^, Disp = 1.95, Quasi-*p* < 0.01, β_int = −0.236 [95% CI_q: −0.406, −0.066]). Additionally, we observed a borderline slightly significant interaction of ffaZOL with the adverse effect of age on the number of dominant follicles (Poisson LR (72): 7.92, *p* < 0.005, Disp = 2.16, Quasi-*p* = 0.0072).

### 2.2. Follicular Fluid MDA Levels

**Figure 2:**
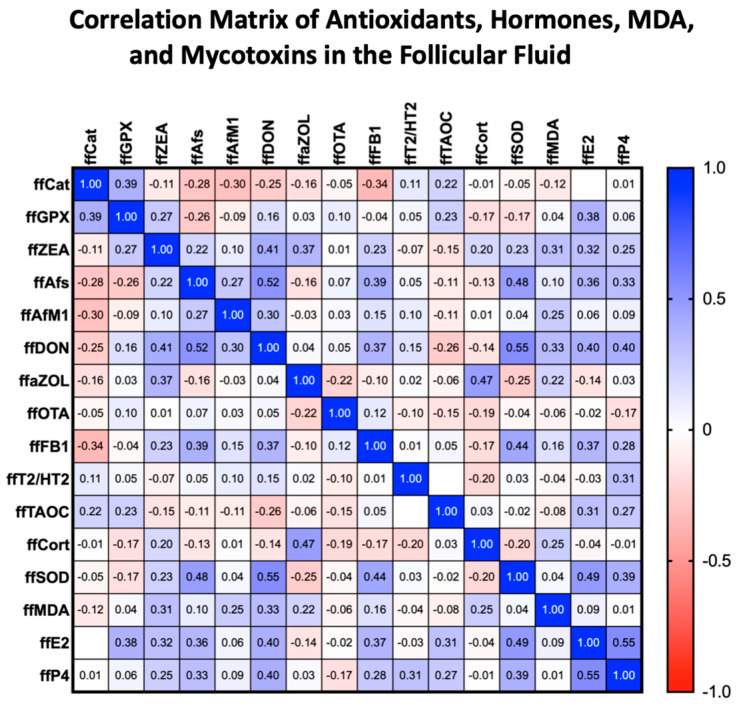
**Correlation Matrix of Antioxidants, Hormones, MDA, and Mycotoxins in the Follicular Fluid.** The table shows positive and negative correlations between the concentrations of mycotoxins, hormones, MDA, and antioxidants measured in the follicular fluid. All measured concentrations were standardized to protein levels. The Spearman coefficient (r) values are displayed in the cells. Positive correlations are highlighted in blue, while negative correlations are highlighted in red. Empty cells indicate that Spearman’s r coefficient is less than 0.01. The intensity of the colors reflects the strength of the correlation.

**Figure 3:**
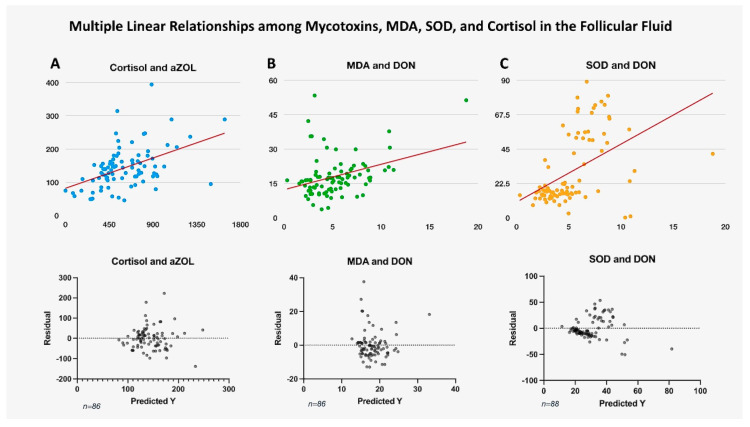
**illustrates multiple linear relationships among mycotoxins, MDA, SOD, and cortisol in follicular fluid**. The figure displays regression relationships along with their residual plots. A positive linear relationship is observed between (**A**): Cortisol and aZOL, (**B**): MDA and DON, and (**C**): SOD and DON. The trend lines are highlighted in red. The bottom row presents the residual plots from the linear regression corresponding to the top images. Due to outliers and variations in standard deviations observed in the figures, transformation models were also applied to prevent statistical bias.

ffMDA concentration positively correlated with DON (r (88) = 0.3501, 95% CI [0.1518 to 0.5213], *p* < 0.001), ffZEA (r (88) = 0.3691, 95% CI [0.1730 to 0.5370], *p* < 0.001), and ffaZOL (r (88) = 0.223, 95% CI [0.08891 to 0.4733], *p* < 0.05) levels (Figure 2). Follicular fluid DON levels showed a weak but positive linear regression relationship (F (1, 86) = 12.07, 95% CI [0.4722 to 1.743], *p* < 0.001, Figure 3). We found no correlation between the other mycotoxins measured in the follicular fluid and ffMDA. There was no significant correlation between the number of follicles and the number of mature follicles. The number of oocytes and the ratio of retrieved oocytes to total follicles showed a negative trend with the measured MDA concentration; however, these values did not reach statistical significance (*p* = 0.085 and *p* = 0.072, respectively). There was no significant correlation between E2, P4, and MDA levels in the follicular fluid.

### 2.3. Serum and Follicular Fluid Cortisol Levels

Follicular cortisol levels correlated with serum cortisol levels (Table 2). However, higher concentrations were found in follicular fluids compared to serum total cortisol levels (*p* < 0.0001), with no significant difference in cortisol levels. No correlation existed between serum and follicular fluid cortisol regarding follicle number or the count of retrieved oocytes. A very strong, positive linear relationship was observed between follicular cortisol and aZOL (F (1, 86) = 25.09, 95% CI [number_1 to 0.1416], *p* < 0.0001). This relationship remained significant after adjusting for the False Discovery Rate (FDR). We also found a weaker but significant positive correlation with MDA levels (F (1, 86) = 9.866, 95% CI [0.8178 to 3.638], *p* < 0.01, Figure 3). Simultaneously, a negative correlation was identified between cortisol and SOD levels (F (1, 86) = 6.145, 95% CI [−1.367 to −0.1349], *p* < 0.05) in the follicles. Additionally, follicular cortisol levels showed a negative correlation with E2 concentrations (F (1, 86) = 9.202, *p* < 0.01). The above correlations lost significance after applying FDR correction. The effect of ffaZOL on cortisol levels depends on the estrogen levels in the follicular fluid. At low estrogen levels, ffaZOL is associated with lower follicular cortisol; however, at high estrogen levels, this effect reverses, correlating with higher cortisol levels. This “opposite-direction effect” results from a significant ffaZOL and ffE2 interaction (F (1, 84) = 36.37, 95% CI [4.566 × 10^−6^ to 9.058 × 10^−6^], *p* < 0.0001). No correlation was found between serum cortisol and mycotoxin levels. A significant positive association was observed between follicular cortisol and the number of usable embryos (β = 0.00399, 95% CI [0.0003 to 0.0047], *p* = 0.005); however, this relationship weakened after applying a negative binomial transformation.

### 2.4. Follicular Fluid SOD Levels

The analysis using multiple linear regression and generalized linear models with a gamma distribution identifies significant predictors of ffSOD, notably ffDON, which shows the strongest significance and a positive estimate (F (1, 88) = 26.48, 95% CI: 2.866 to 6.493, *p* < 0.0001, Figure 3). This suggests that increases in ffDON are linked to increases in ffSOD. Other variables, such as ffAfM1 (F (1, 88) = 7.013, *p* < 0.01), act as negative predictors, while ffE2 (F (1, 88) = 5.708, p < 0.05) functions as a positive predictor, both with significant but small effects. Additionally, no correlation was found between SOD levels and follicle numbers, retrieved oocytes, or the number of embryos. There was also no correlation between SOD and MDA levels in follicular fluid.

### 2.5. Total Antioxidant Capacity, CAT Activity, and GPx Activity

**Figure 4:**
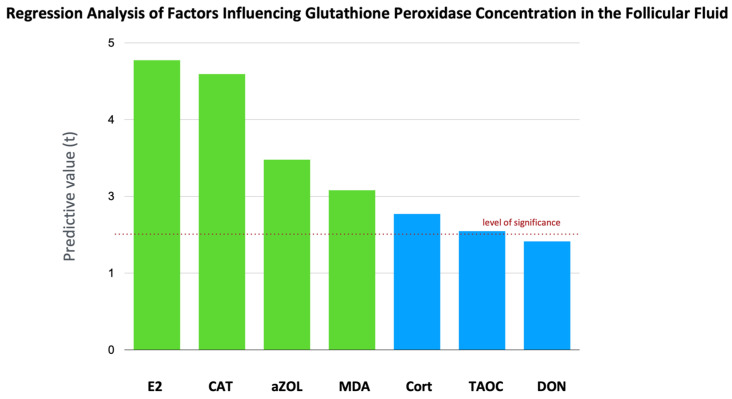
**Regression analysis of factors influencing GPx concentration in follicular fluid.** The level of GPx in follicular fluid shows a strong positive relationship with ffE2 (F (1, 30) = 22.25, *p* < 0.0001), ffMDA (F (1, 30) = 6.755, *p* < 0.05), and ffCAT (F (1, 30) = 20.18, *p* < 0.0001). The level of ffaZOL also has a weak positive effect (F (1, 30) = 9.594, *p* < 0.01). We found a negative regression relationship with cortisol (F (1, 30) = 4.897, *p* < 0.05). In this model, a multivariate regression including several predictors explained 62.96% of the variability in GPx. The results showed that the negative effects of DON and TAOC were at trend level (*p* = 0.0846, *p* = 0.0629), likely due to the relatively small sample size. The green color indicates a positive effect, while the blue color indicates a negative effect.

The level of CAT activity showed significant negative associations. There is a negative regression relationship for AfM1 (F (1, 86) = 5.027, *p* < 0.05), DON (F (1, 86) = 4.543, *p* < 0.05), and FB1 (F (1, 86) = 9.958, *p* < 0.01, Figure 2). Based on our results, it appears that in this negative regression, there is an antagonistic effect between DON and FB1 (F (1, 84) = 6.079, CI [“−0.006958 to −0.0007449”], *p* < 0.05). The level of CAT activity shows a positive correlation with the levels of GPx activity and TAOC (r = 0.3901, r = 0.2217, *p* < 0.05, respectively); however, no correlation was found with the levels of SOD, cortisol, and MDA. The level of GPx in follicular fluid shows a strong positive relationship with ffE2 (F (1, 30) = 22.25, *p* < 0.0001), ffMDA (F (1, 30) = 6.755, *p* < 0.05), and ffCAT level (F (1, 30) = 20.18, *p* < 0.0001). The level of ffaZOL also has a weak positive effect (F (1, 30) = 9.594, *p* < 0.01). We found a negative regression relationship with cortisol (F (1, 30) = 4.897, *p* < 0.05). In this model, a multivariate regression including several predictors, which explained 62.96% of the variability in GPx activity (Figure 4), showed that the negative effects of DON and TAOC were at the trend level (*p* = 0.0846, *p* = 0.0629), likely due to the relatively small sample size. The level of TAOC shows the strongest relationship with the levels of E2 and P4 in follicular fluid (F (1, 85) = 15.08, *p* < 0.001), (F (1, 85) = 7.38, *p* < 0.01). The measured TAOC levels did not show significant associations with mycotoxin levels across the tested models.

## 3. Discussion

Similar to many previously published animal experimental results [34,35], our research group was the first to demonstrate the presence of the most commonly occurring mycotoxins in human follicular fluid [6]. In this study, we aimed to determine whether mycotoxin concentrations in human follicular fluid can influence oxidative stress processes that are essential for folliculogenesis and oocyte maturation. Lipid peroxidation and the stress response in follicular fluid were assessed by measuring MDA and cortisol levels. To evaluate antioxidant defense activity, SOD levels were measured in the follicular fluid. Our findings revealed no correlation between age, BMI, AMH, and the levels of MDA, cortisol, and SOD. Other studies have reported similar results [36,37,38], who also found no relationship between physical activity, BMI, age, MDA levels, and total antioxidant capacity.

The data suggest that, although there is generally no direct link between the number of follicles or oocytes retrieved and mycotoxin levels, more subtle interactions are present. For example, we observed a positive correlation between the number of follicles and the mycoestrogen metabolite, ffaZOL. This positive correlation may be related to factors such as follicle stimulation and hormonal status. Anti-Müllerian hormone (AMH) and antral follicle count could be associated with follicular activity and hormonal shifts that also influence mycotoxin metabolite levels. Both AMH and follicle count serve as important predictors of ovarian response and are often tied to hormonal balance dynamics [39,40]. Another explanation may involve the metabolism of follicles and their sensitivity, particularly to external hormonal stimulation, such as follicle-stimulating hormone (FSH) [40,41]. The number of antral follicles is positively related to follicular success and embryo production, indicating that levels of mycotoxin metabolites in follicles may impact follicle development and quantity [39].

Therefore, an increase in follicle number may be linked to higher ffaZOL concentration, reflecting hormonal regulation and metabolic activity during follicular development. Understanding these relationships might enhance knowledge of how mycotoxins affect ovarian function and reproductive health. Although this observation did not reach significance after some statistical corrections, it suggests a potential, yet complex, influence that warrants further investigation. This contrasts with some animal studies where high-dose mycotoxin exposure has been shown to decrease the number of primordial follicles and disrupt ovarian function. One study on weaned gilts found that dietary exposure to ZEA caused a reduction in the thickness of the primordial follicle layer, indicating follicular atresia at high doses [42]. The more subtle trends observed in this study may suggest that chronic, low-dose exposure has different, more complex effects on human reproductive physiology.

The interaction between DON and AMH is important, with lower DON levels boosting the positive effect of AMH on the number of dominant follicles. The interaction between DON and AMH enhances our understanding of how environmental toxins can influence reproductive functions. AMH is a key hormone produced by granulosa cells in developing ovarian follicles, playing a vital role in both the recruitment of primordial follicles and the selection of dominant follicles [43,44]. Research indicates that AMH’s expression peaks in smaller antral and preantral follicles and gradually decreases as follicles mature, suggesting its role in regulating early follicular development [44,45].

DON, a mycotoxin, is known to impact cellular functions by disrupting protein synthesis and inducing oxidative stress [46]. DON can enhance the estrogen signaling pathway induced by E2 or ZEN [47].

When DON levels are lower, they may not significantly interfere with AMH’s role, allowing AMH to support normal follicular development and enhance the positive effect on dominant follicle numbers. The combined influence of lower DON levels and AMH could therefore promote the proper selection of dominant follicles by decreasing the inhibitory effects of increased oxidative stress typically associated with high DON exposure. This interaction highlights the potential of AMH to regulate follicle development even under conditions that might otherwise disrupt normal reproductive processes. Although further research is needed to clarify the exact mechanisms, the interaction between lower DON levels and AMH likely reflects a balance in which AMH maintains its regulatory function within ovarian physiology. This is especially important since AMH is a key biomarker for assessing ovarian reserve. Higher DON levels may impair the beneficial role of AMH, possibly through mechanisms that are not yet fully understood, but may involve the disruption of hormonal signaling or induction of cellular stress, thereby affecting AMH production.

### 3.1. The Link with Oxidative Stress

The results show a clear link between mycotoxins and markers of oxidative stress in follicular fluid. MDA (malondialdehyde), a marker of lipid peroxidation and oxidative damage, is positively associated with several mycotoxins, including ffDON, ffZEA, and ffaZOL [48]. ZEA and its metabolite alpha-ZOL can alter metabolic processes, leading to oxidative stress and changes in systemic metabolism, which affect cell membrane integrity and protein synthesis. These disruptions can contribute to impaired ovarian function and follicular development [49]. This close relationship supports the notion that mycotoxins contribute to the promotion of oxidative stress in the follicular microenvironment. Oxidative stress processes occur to varying degrees at different stages of folliculogenesis. It helps initiate angiogenesis and apoptosis of other accessory follicles to assist in selecting the dominant follicle, while a significant increase in oxidative stress can have harmful effects [15]. Increased reactive oxygen species in the follicular environment, leading to mitochondrial damage, reduced mtDNA copy number, and compromised oocyte bioenergetics—effects that impair oocyte developmental competence even when follicle numbers remain unchanged [50,51].

### 3.2. The Antioxidant Counterbalances

Analysis of antioxidant enzymes provides further insight. SOD (superoxide dismutase), a key antioxidant enzyme [9], was positively correlated with DON levels in our recent study, indicating that the body responds to oxidative stress caused by mycotoxins. This is reinforced by the positive correlation between ffSOD and ffE2, as it is known [23] that SOD levels in follicular fluid help create a more stable environment for follicular cells, including granulosa and theca cells, which are essential for steroidogenesis and produce E2 [23]. The interaction between ffDON, ffSOD, ffAfM1, and ffE2 in predicting biological outcomes can be complex due to their varying roles as predictors. When levels of ffDON increase, ffSOD levels also tend to rise, which may suggest a compensatory response to oxidative stress, as superoxide dismutase is an antioxidative enzyme. This relationship implies a biological mechanism where increased low-concentration toxin levels, such as ffDON, could induce a protective oxidative stress response, reflected by elevated ffSOD activity.

ffAfM1 serves as a negative predictor with a significant value, indicating that higher levels of ffAfM1 are linked to a negative outcome, likely due to its known harmful effects, such as causing liver damage or acting as a carcinogen. Meanwhile, ffE2 functions as a positive predictor, suggesting that it has beneficial roles, possibly related to its natural presence in biological systems that positively influence physiological processes.

The context indicates that although both ffAfM1 and ffE2 have significant predictive abilities, their effect sizes are smaller compared to ffDON, emphasizing ffDON’s prominent role in influencing these biological processes. This study also showed a positive correlation between GPX activity (glutathione peroxidase) and ffaZOL, suggesting that the body works to counteract the harmful effects of mycotoxin exposure [9]. Conversely, the inverse relationship between catalase and mycotoxins [52], such as DON, AfM1, or FB1, may indicate that these toxins inhibit the function of this essential antioxidant enzyme. These complex and sometimes conflicting relationships highlight the multifaceted ways mycotoxins disrupt the oxidative balance of follicular fluid.

Exposure to low levels of DON in follicular fluid can trigger the production of ROS, which then increases MDA levels, signaling heightened lipid peroxidation. The body often responds to elevated oxidative stress by boosting the activity of antioxidant enzymes, such as SOD, to neutralize ROS and safeguard cellular structures from damage [53,54]. In follicular fluid, this oxidative stress and the body’s response can impair follicle quality and ovarian function by damaging cellular components and impacting signaling pathways like JNK/c-Jun that regulate apoptosis and stress responses [55]. Therefore, the concurrent rise in MDA and SOD levels in follicular fluid upon DON exposure reflects increased oxidative damage, along with the cellular antioxidant system’s effort to mitigate this stress.

The lack of a significant correlation between MDA and oocyte or embryo count in this data may be due to other repair mechanisms and/or the relatively small sample size. However, the connection between mycotoxins and oxidative stress remains clearly evident.

### 3.3. Endocrine Disruptor Effects

Hormonal interactions: the significance of ffE2 and ffcortisol.

This study revealed significant interactions between mycotoxins and hormones, especially cortisol and estradiol (E2). Follicular cortisol levels exceeded serum levels, supporting Andersen’s (2002) [56] earlier finding that follicular fluid contains enzymes, such as 11 beta-hydroxysteroid dehydrogenase, which can convert cortisone, an inactive form of cortisol, back into active cortisol, thereby increasing its local concentration and activity within the follicle. This conversion allows cortisol to potentially regulate processes such as inflammation linked to ovulation [56]. A strong, positive linear relationship was observed between follicular free cortisol and aZOL. Additionally, the impact of ffaZOL on follicular cortisol depended on estrogen levels—at low estrogen, ffaZOL was associated with decreased cortisol, while at high estrogen, this effect was reversed. Lower follicular cortisol levels, coupled with reduced estrogen, suggest a hormonal imbalance. Estrogen deficiency may lead to decreased sensitivity or suppression of cortisol, resulting in lower cortisol levels [57]. Since cortisol plays a crucial role in the timing of egg maturation and ovulation [58], it is reasonable to assume that influencing follicular cortisol levels may also change the timing of ovulation. Too early or even delayed ovulation, along with local hormonal imbalance, can cause asynchrony with the implantation window period [59,60,61,62].

This uncovers a new, biologically based interaction, where mycotoxins like ZEN or DON act as endocrine disruptors, altering key hormone functions depending on environmental conditions. DON amplifies the estrogen signaling pathway triggered by E2 or ZEN [47]. This aligns with previous research indicating that mycotoxins—especially zearalenone and its derivatives—have estrogen-like properties [8,63,64]. Furthermore, the findings suggest that mycotoxins contribute to oxidative stress and disrupt endocrine balance, affecting hormonal and antioxidant functions that are crucial for healthy follicular development and oocyte quality. The complex interactions, such as the modulation of AMH by DON and the estrogen-dependent effect of ffaZOL on cortisol, highlight the need for further studies to fully understand how these environmental pollutants influence human reproduction.

## 4. Conclusions

This study shows that mycotoxins in follicular fluid can significantly increase oxidative stress and disrupt the antioxidant balance, regardless of factors like age, BMI, and cortisol levels. Notably, certain mycotoxins (ffDON, ffZEA, and ffaZOL) are positively linked to markers of oxidative stress, while SOD levels are positively associated with DON and E2. Therefore, the simultaneous rise in MDA and SOD levels in follicular fluid after DON exposure indicates increased oxidative damage, along with the cellular antioxidant system’s effort to counter this stress. However, no clear connections were found between mycotoxin levels and reproductive outcomes such as follicle count or retrievable embryos. These findings suggest that even low levels of mycotoxin exposure can harm follicular health and oocyte quality. Consequently, this study recommends stricter food safety regulations and better monitoring to reduce mycotoxin contamination, especially among vulnerable populations. Further research is needed to better understand the impact of mycotoxins on reproductive health and oxidative stress mechanisms.

Limitations: This study used data from patients undergoing IVF treatment. This group is diverse in terms of their diet, potential comorbidities, and the causes of infertility. Since a healthy control group was not feasible due to ethical, methodological, and testing reasons, only cautious conclusions can be drawn from these results regarding acceptable mycotoxin levels from a health perspective. The primary aim of our study was to explore the relationships between relatively low doses of mycotoxins and altered physiological processes in the follicle.

## 5. Materials and Methods

### 5.1. Patients

Patients with infertility who underwent controlled gonadotropin stimulation were included in this study. They received treatment and had oocytes retrieved between June 2024 and May 2025. After receiving detailed verbal and written information and signing a consent form approved by the regional ethics committee (SE-RKEB 86/2023), 88 patients participated. During the cycle, serum hormone tests were performed on days 2–4 and 12, measuring FSH, LH, and E2. AMH level was measured in the previous cycles during the examination period. Before egg retrieval, a transvaginal ultrasound was used to measure visible follicles accurately. Oocyte retrievals took place at the Center of Assisted Reproduction, Department of Obstetrics and Gynecology, Semmelweis University, Budapest. On the day of retrieval, blood samples were retaken to assess serum mycotoxin levels, followed by follicle aspiration and oocyte retrieval under surgical conditions. Post-isolation, follicular fluid was sampled and centrifuged. Any follicular fluid visibly contaminated with blood was excluded. The remaining follicular fluid from all follicles was pooled, as individual collection increases intervention and blood contamination risks [34]. Serum and follicular samples were stored at −70 °C.

### 5.2. Mycotoxin Analyses

Mycotoxin quantification was conducted on both follicular fluid and plasma samples. The analytical procedures employed were those previously delineated in our earlier publications [2,6].

### 5.3. Steroid Analyses

To analyze sex steroids in serum and follicular fluid, kits for 17-beta-estradiol (Cat No: DNOV003, NovaTec Immundiagnostica, Dietzenbach, Germany) and progesterone (Cat No: DNOV006, NovaTec, Dietzenbach, Germany) were used [6]. For cortisol (Cat No: DNOV001, Gold Standard Diagnostic, Dietzenbach, Germany) analysis in serum and follicular fluid, an immunoassay was performed according to the manufacturer’s instructions. All samples were measured in triplicate. Data were collected and analyzed using a Thermo MultiskanTM FC microplate reader (Waltham, MA, USA) using SkanIt RE software (version 6.1.1.7). Absorbance was measured at 450 nm, with a reference at 630 nm.

### 5.4. Serum Anti-Müllerian Hormone (AMH) Measurements

The anti-Müllerian hormone (AMH) was measured using the Beckman Coulter Access 2 Immunoassay System from California City, CA USA. This was carried out with a chemiluminescence immunoassay (CLIA) method. The Access AMH Advanced test (Cat. No: B13127) uses a special particle and light-based test to measure AMH levels in human blood. The tests were conducted twice, following the manufacturer’s instructions [6].

### 5.5. FF Protein Measurements by Nanodrop

The protein concentration of follicular fluid samples was measured using a Nanodrop (Thermo Fisher Scientific, Verona, WI, USA). The samples were analyzed on Protein A280 Setup utilising BSA (bovine serum albumin) as the standard. The baseline correction was set at 340 nm. Bovine serum albumin served as the reference. Unknown (simple) protein concentrations were calculated using the mass extinction coefficient of 6.7 at 280 nm for a 1% (10 mg/mL) BSA solution. 0,01 M PBS (phosphate-buffered saline) was employed as the blank. The measurements were performed in quintuplicate.

### 5.6. SOD Analysis in Follicular Fluid

The Invitrogen Superoxide Dismutase (SOD) Colorimetric Activity Kit, Catalog Number EIASODC, was utilised for FF SOD analyses.

Follicular fluid samples were diluted 1:5 with 1X Assay Buffer. Then, 10 μL of the diluted samples was added to the microplate wells. In addition, 50 μL of 1X Substrate was subsequently added to each well. Twenty-five microL of 1X Xanthine Oxidase initiated the colorimetric reaction. The plate was incubated at room temperature for 20 min. Absorbance was measured at 450 nm using a microplate reader. A blanking procedure was performed if samples exhibited a yellow colour, subtracting pre-Xanthine Oxidase absorbance. This assay quantified total Superoxide Dismutase (SOD) activity. Standard safety protocols, including the use of personal protective equipment (PPE), were strictly adhered to. The results reflect the SOD activity within the follicular fluid.

The FF samples and standards were pipetted into a 96-well plate, and colorimetric measurements were taken using a Thermo MultiskanTM FC (Waltham, MA, USA) equipped with SkanIt RE software (version 6.1.1.7). Absorbance readings were recorded at a wavelength of 450 nm. Data analysis was performed using the SkanIt RE software (version 6.1.1.7).

### 5.7. Glutathione Peroxidase (GPx) Activity Assay

Glutathione peroxidase (GPX) activity in follicular fluid samples was measured using the Glutathione Peroxidase Assay Kit (Catalog Number MAK437, Sigma-Aldrich Darmstadt, Germany). GPX activity was determined by monitoring the consumption of NADPH at 340 nm in a 96-well plate using a Thermo MultiskanTM FC (Waltham, MA, USA) equipped with SkanIt RE software (version 6.1.1.7). The reaction was initiated by the addition of peroxide solution, and the decrease in optical density over time, directly proportional to GPX activity, was measured. A standard curve was generated using NADPH standards, and GPX activity was expressed in units per liter (U/L).

### 5.8. Catalase Activity Assay

Catalase activity in follicular fluid samples was measured using the Catalase Colorimetric Activity Kit (Catalog Number EIACATC, Invitrogen, Waltham, MA, USA). The ff samples were lysed using 1X Assay Buffer. The supernatant was collected and diluted in 1X Assay Buffer. Samples and catalase standards were added to a 96-well microplate, followed by Hydrogen Peroxide Reagent. After a 30 min incubation, Substrate and 1X HRP solution were added. The absorbance was measured at 560 nm using a Thermo MultiskanTM FC (Waltham, MA, USA) microplate reader. Catalase activity was determined by comparing the sample absorbance to a standard curve generated with known catalase concentrations.

### 5.9. FF MDA Assay

The assessment of lipid peroxidation was conducted by measuring malondialdehyde (MDA) concentrations in follicular fluid using the Thiobarbituric Acid Reactive Substances Assay. The measurements were performed using a Lipid Peroxidation (MDA) Assay Kit (MAK085, Sigma-Aldrich, Merck, Darmstadt, Germany). The assay was executed following the manufacturer’s protocol. In this process, MDA in the FF sample combines with thiobarbituric acid (TBA) to form an MDA-TBA complex, which is then quantified through colorimetric analysis. For the colorimetric test, a 0.1 mol/L MDA standard was created, and a series of dilutions was prepared to establish a standard curve. The FF samples and standards were pipetted into a 96-well plate, and colorimetric measurements were taken using a Thermo MultiskanTM FC (Waltham, MA, USA) equipped with SkanIt RE software (version 6.1.1.7). Absorbance readings were recorded at a wavelength of 532 nm. The absorbance value of the blank (MQ water) was subtracted to account for background interference. Data analysis was performed using the SkanIt RE software (version 6.1.1.7).

### 5.10. Total Antioxidant Capacity (T-AOC) Assay

The total antioxidant capacity (T-AOC) in follicular fluid was determined using the Total Antioxidant Capacity Colorimetric Assay Kit (ABTS, Enzyme Method) (Catalog No. EEA023, Invitrogen). Briefly, follicular fluid samples were prepared according to the manufacturer’s instructions. ABTS working solution was prepared by mixing buffer solution, ABTS solution, and H_2_O_2_ application solution (152:10:8). Peroxidase application solution was prepared by mixing buffer solution and peroxidase (9:1). Samples and Trolox standards were added to a 96-well microplate, followed by peroxidase application solution and ABTS working solution. The absorbance was measured at 414 nm using a Thermo MultiskanTM FC (Waltham, MA, USA) microplate reader after a 6 min incubation at room temperature. T-AOC was calculated using a standard curve generated with Trolox standards.

### 5.11. Statistical Analysis

Descriptive statistics were computed for all variables. Normality of continuous data was evaluated using the D’Agostino-Pearson, Shapiro–Wilk, Anderson-Darling, and Kolmogorov–Smirnov tests. For correlation analyses, Pearson’s correlation coefficient was applied for normally distributed variables, while Spearman’s rank correlation and log1p-transformed Ordinary Least Squares (OLS)/Gamma Generalized Linear Model (GLM) log link sensitivity were used when normality assumptions were not met. A correlation matrix was generated to explore relationships among key variables. To assess the impact of clinical and biochemical factors on outcome measures, multiple linear regression, Poisson, and Quasi-Poisson regression models were employed as appropriate. Linear models were used when assumptions of normality and homoscedasticity were satisfied; otherwise, generalized linear models with a Poisson distribution were utilized. To reduce potential statistical bias and false positives, in cases of non-normal distributions, transformation models were also applied. Multicollinearity was checked with variance inflation factors (VIF), with a VIF greater than 5 indicating potential multicollinearity. Variance inflation was considered acceptable when it was below 1.5. Model fit was evaluated using R^2^ or pseudo-R^2^ values, as well as residual diagnostics. A *p*-value of less than 0.05 was deemed statistically significant. All statistical analyses were conducted using IBM SPSS Statistics Premium 30 (Chicago, IL, USA, GraphPad Prism 10 (version 10.5.0, San Diego, CA, USA), and MATLAB (R2022b, The MathWorks Inc., Natick, MA, USA).

## Figures and Tables

**Table 1:** **Biophysical, hormonal, and follicular maturation parameters** of eighty-eight patients who underwent IVF treatment. The table shows the mean, median, standard deviation, as well as minimum and maximum values of the primary biophysical and hormonal parameters. It also provides descriptive statistics on folliculogenesis, oocytes, and usable embryos. ^⊘^ The diameter of follicles. ^✴^ The follicular level is significantly higher than serum. ^†^ Serum and follicular levels are correlated. The Mann–Whitney nonparametric U-test and Spearman correlation were used.

	Mean	Standard Deviation	Median	Minimum	Maximum
Age	36.44	4.276	37.0	28	44
BMI	24.62	4.498	23.8	17.6	37.7
Anti-Mullerian Hormone (μg/L)	2.092	1.999	1.450	0.15	8.53
Total number of Follicles (n)	10.5	5.543	10	2	27
Dominant Follicles (^⊘^ > 15 mm, n)	7.641	4.385	7	1	21
Useable Embryos (n)	1.942	1.929	1	0	9
Ratio of oocytes/all follicles	0.41	0.41	0.4	0	1.17
Ratio of embryos/all follicles	0.13	0.13	0.09	0	0.6
Ratio of embryos/dominant follicles	0.18	0.2	0.13	0	0.9
Serum P4 (μg/L)	1.438 ^†^	0.9248	1.2	0.4	5.1
Ff P4 (μg/L)	6846 ^✴,†^	2144	6274	1500	12,966
Serum E2 5.day (pg/ml)	48.01	17.92	48.0	17.1	84.7
Serum E2 12.day (pg/ml)	1811	1207	1504	295	4575
Ff E2 (pg/ml)	48,101 ^✴^	13,720	46,460	26,861	108,050
FSH (IU/L)	6.401	3.087	6.1	0.8	17.0
LH (IU/L)	5.833	2.747	5.3	1.2	14.4

**Table 2:** **Serum and follicular mycotoxin levels.** The table presents the mean, median, standard deviation, and the minimum and maximum concentrations of mycotoxins in serum and follicular fluid standardized to protein levels. ^✴^ The follicular mycotoxin level is significantly higher than in serum. ^†^ The levels in serum and follicular fluid are correlated. The Mann–Whitney nonparametric U-test and Spearman correlation were used.

	Mean	Standard Deviation	Median	Median in nM	Minimum	Maximum
serum Aflatoxin (pg/ml)	1.364	7.423	0	0	0	47
Ff Aflatoxin (pg/ml)	27.92	73.31	0	0	0	432.7
serum Aflatoxin M1 (pg/ml)	11.5 ^†^	19.59	5.258	1.68 × 10^−5^	0	133.5
Ff Aflatoxin M1 (pg/ml)	11.49 ^†^	18.14	6.12	1.86 × 10^−5^	0	114.1
serum Zearalenone (pg/ml)	66.18	78.38	44.44	1.39 × 10^−4^	0	391.19
Ff Zearalenone (pg/ml)	155.5 ^✴^	239.2	99.1	3.11 × 10^−4^	0	1744
serum alpha-Zearalenol (pg/ml)	129.5 ^†^	105.9	107	3.33 × 10^−4^	0	438
Ff alpha-Zearalenol (pg/ml)	605.4 ^✴,†^	290.9	543.1	1.69 × 10^−3^	0	1637
serum Ochratoxin A (pg/ml)	9.659	9.189	8	1.98 × 10^−5^	0	60
Ff Ochratoxin A (pg/ml)	9.62	8.84	6	1.48 × 10^−5^	0	37.33
serum Fumonisin B1 (pg/ml)	70.84 ^†^	99.78	24.4	3.38 × 10^−4^	0	388.4
Ff Fumonisin B1 (pg/ml)	53.37 ^✴,†^	86.26	0	0	0	405.3
serum Deoxynivalenol (ng/ml)	3.47	2.98	2.69	9.07 × 10^−3^	0	19.35
Ff Deoxynivalenol (ng/ml)	5.26 ^✴^	2.82	4.80	1.61 × 10^−2^	0.31	18.77
serum T2/HT2 toxin (ng/ml)	1.90 ^†^	1.145	2.13	4.56 × 10^−3^	0	4.25
Ff T2/HT2 toxin (ng/ml)	1.86 ^†^	1.39	1.88	4.02 × 10^−4^	0	7.82

## Data Availability

The data presented in this study are available upon request from the corresponding author due to restrictions concerning the protection of privacy and ethical considerations.

## Abbreviations

The following abbreviations are used in this manuscript:

DON	Deoxynivalenol
ZEN	Zearalenone
aZOL	Alpha-zearalenol
AfM1	Aflatoxin M1
MDA	Malondialdehyde
SOD	Superoxide dismutase
GPx	Glutathione peroxidase
CAT	Catalase
TAOC	Total antioxidant capacity
AMH	Anti-Müllerian hormone
WHO	World Health Organization
OTA	Ochratoxin A
ROS	Reactive oxygen species
AFs	Aflatoxins
FB1	Fumonisin B1
AOH	Alternariol
BMI	Body mass index
IVF	In vitro fertilization
E2	Estradiol
P4	Progesterone
FSH	Follicle-stimulating hormone
FDR	False Discovery Rate
JNK	c-Jun N-terminal kinase
